# Harnessing technology and gamification to increase adult physical activity: a cluster randomized controlled trial of the Columbia Moves pilot

**DOI:** 10.1186/s12966-023-01530-1

**Published:** 2023-11-03

**Authors:** Courtney M. Monroe, Bo Cai, Sarah Edney, Danielle E. Jake-Schoffman, Keith Brazendale, Agnes Bucko, Bridget Armstrong, Chih-Hsiang Yang, Gabrielle Turner-McGrievy

**Affiliations:** 1 Arnold School of Public Health, Department of Health Promotion, Education, and Behavior, University of South Carolina, Discovery 1 Building, Suite 403G, 915 Greene Street, Columbia, SC 29208 USA; 2 Arnold School of Public Health, Department of Epidemiology and Biostatistics, University of South Carolina, Discovery 1 Building, Room 460, 915 Greene Street, Columbia, SC 29208 USA; 3 Saw Swee Hock School of Public Health, National University of Singapore, Tahir Foundation Building (Block MD1), 12 Science Drive 2, #11-01, Singapore, 117549 Singapore; 4 College of Health and Human Performance, Department of Health Education and Behavior, University of Florida, P.O. Box 118210, Gainesville, FL 32611 USA; 5 College of Health Professions and Sciences, Department of Health Sciences, University of Central Florida, HS II, Room 210A, 12805 Pegasus Drive, Orlando, FL 32816 USA; 6 College of Health and Human Services, University of North Carolina-Charlotte, 8844 Craver Road, Charlotte, NC 28223 USA; 7 Arnold School of Public Health, Department of Exercise Science, University of South Carolina, Public Health Research Center, Room 132, 921 Assembly Street, Columbia, SC 29208 USA; 8 Arnold School of Public Health, Department of Exercise Science, University of South Carolina, Discovery 1 Building, Room 403E, 915 Greene Street, Columbia, SC 29208 USA; 9 Arnold School of Public Health, Department of Health Promotion, Education, and Behavior, University of South Carolina, Discovery 1 Building, Room 552, 915 Greene Street, Columbia, SC 29208 USA

**Keywords:** Physical activity, Gamification, Social support, e/mHealth

## Abstract

**Background:**

The use of health technologies and gamification to promote physical activity has increasingly been examined, representing an opportunistic method for harnessing social support inherent within existing social ties. However, these prior studies have yielded mixed findings and lacked long-term follow-up periods. Thus, a pilot cluster randomized controlled trial was conducted to gauge the feasibility and preliminary efficacy of a digital gamification-based physical activity promotion approach among teams of insufficiently active adults with existing social ties.

**Methods:**

Teams (*N* = 24; 116 total participants) were randomized to either a 12-week intervention (Fitbit, step goals, app, feedback; TECH) or the same program plus gamification (TECH + Gamification). Mixed effects models were used to compare group differences in treatment adherence, and changes in social support, steps, and moderate-to-vigorous physical activity at 12 weeks and 52 weeks from baseline, adjusted for sociodemographic characteristics and team size.

**Results:**

TECH had a lower mean number of days of Fitbit self-monitoring versus TECH + Gamification during the intervention (adjusted difference: -.30; 95% CI, -.54 to -.07; *P* = .01). Post-intervention, TECH had 47% lower odds of self-monitoring 7 days per week versus TECH + Gamification (.53; 95% CI, .31 to .89; *P* = .02). No differences were observed between TECH + Gamification and TECH in increases in social support (0.04; 95% CI, -.21 to .29; *P* = .76), ActiGraph-measured daily steps (-425; 95% CI, -1065 to 215; *P* = .19), or moderate-to-vigorous physical activity minutes (-3.36; 95% CI, -8.62 to 1.91; *P* = .21) from baseline to 12 weeks or in the regression of these improvements by 1 year (*Ps* > .05). Although not significant in the adjusted models (*Ps* > .05), clinically meaningful differences in Fitbit-measured daily steps (TECH, 7041 ± 2520; TECH + Gamification, 7988 ± 2707) and active minutes (TECH, 29.90 ± 29.76; TECH + Gamification, 36.38 ± 29.83) were found during the intervention.

**Conclusions:**

A gamified physical activity intervention targeting teams of adults with existing social ties was feasible and facilitated favorable, clinically meaningful additive physical activity effects while in place but did not drive enhanced, long-term physical activity participation. Future investigations should explore optimal team dynamics and more direct ways of leveraging social support (training teams; gamifying social support).

**Trial registration:**

Clinicaltrials.gov (NCT03509129, April 26, 2018).

**Supplementary Information:**

The online version contains supplementary material available at 10.1186/s12966-023-01530-1.

## Background

The 2018 Physical Activity (PA) Guidelines for Americans recommend achieving 150 min or more per week of moderate-intensity equivalent PA – which translates to approximately 7,000–8,000 steps or more per day [1] – to obtain numerous physical and mental health benefits, recognizing that more is better [2, 3]. Yet, over half of U.S. adults are insufficiently physically active [2, 4, 5], leading to rising health care expenditures and an increased chronic disease risk [2, 6, 7]. Theory-based interventions, including those incorporating smartphones or wearable PA trackers for self-monitoring step counts (a cornerstone digital PA behavior change technique) [8, 9], can effectively foster the initiation of a physically active lifestyle [9–11]; however, they frequently fail to facilitate long-term maintenance of initial PA improvements [12–15]. There is a persistent public health need to identify scalable PA interventions which will have an enhanced and durable impact at the population level [10, 11, 16, 17].

Ecological models [18, 19] and the Community Preventive Services Task Force [20] endorse interventions that focus on strengthening and maintaining existing social relationships that provide support for PA behavior change. Considerable epidemiological evidence conducted among adults points to a favorable link between social support from existing social ties and leading a physically active lifestyle [21–28]. The potential impact of social support to catalyze lasting physical activity behavior improvements may be amplified within existing social relationships due to the propensity of individuals to attach meaning to their close social ties and seek proximity to them in times of need [22, 28, 29]. However, experimental evidence focused on mediators of behavior change maintenance in PA interventions remains equivocal [30]. Many PA interventions have primarily focused on the individual by conveying the value of seeking social support or attempting to foster it among persons previously unacquainted with one another, potentially helping explain the inconsistency in findings [20, 31–34]. An increasing number of PA interventions have directly engaged the existing social context and demonstrated promise for promoting favorable short-term outcomes [35–55]; yet, these studies were limited by a lack of randomization, device-based PA measures, long-term follow up, and/or scalability concerns [35–55]. Thus, important questions remain about best practices for facilitating maintenance of initial PA increases via the direct targeting of existing social contexts.

Gamification [56] offers an attractive method for harnessing the influence of existing social relationships for improved PA by seeking to promote positive social interactions and openness to positive behavioral influences [57, 58]. It is characterized by the influence motivational affordances (i.e., components and mechanics that structure games) have on psychological outcomes and experiences, and in turn, motivation and behavioral outcomes [59]. Popular commercially available electronic and mobile health (e/mHealth) technologies [60, 61] allow for the seamless delivery of dynamic and interactive gamification strategies (e.g., challenges, competitions, rewards, etc.) within one’s natural context with little to no added burden with respect to materials and time constraints, fueling a rapid growth in gamified commercial apps [62] and PA interventions with the potential for ready scalability [63–65]. Recent reviews centered on the effectiveness of gamification interventions for increasing PA have revealed mixed findings [63–65]. Further, only a small number of prior gamification-based PA interventions have isolated the effect of a given social incentive, team-based gamification approach [37, 40, 53, 66–73]. Of those, fewer than half were conducted in the U.S [37, 66, 70, 72, 73], and just one PA promotion gamification intervention carried out among adults spanned 1 year [66], prompting a continued call for the conduction of rigorous randomized controlled trials (RCTs) that test novel ways to create sustained intervention effects [63–65].

Thus, the purpose of this cluster pilot RCT was to evaluate the feasibility (treatment adherence and satisfaction) and preliminary efficacy of an e/mHealth gamification approach designed to harness the influence of existing social ties for increasing steps and moderate-to-vigorous physical activity (MVPA) among insufficiently physically active adults.

## Methods

### Study design

A 12-week (April-July 2018), parallel-group, pilot cluster RCT for promoting PA called Columbia Moves was conducted. Participants were recruited (January-April 2018) from the Greater Columbia, South Carolina, U.S.A. area via flyers, e-mails sent via listservs, and word of mouth. Interested individuals were encouraged to form a self-selected team of 3–8 persons comprised of members in their existing social circle (e.g., friends, family members, co-workers). Individuals applied through a study recruitment website and were screened for eligibility by phone. Then, they were invited to an in-person orientation and informed consent was obtained. Following the orientation session, individuals’ PA status was measured via the ActiGraph GT9X Link accelerometer (ActiGraph, Pensacola, FL), serving both as confirmation of eligibility and a baseline measure. Additional baseline assessments occurred at a subsequent visit. Treatment assignment was then communicated to each eligible team of individuals via email and an in-person kickoff meeting was administered to introduce them to their respective intervention. Assessments were conducted immediately after the 12-week intervention and at 40 weeks post-intervention. The assessor was not blind to group allocation, but assessment instructions were standardized, the PA measures were device-based, and survey items were administered online via LimeSurvey, minimizing the risk of assessor bias. Incentives were offered for completing these two assessment periods (drawing for a $50 gift card for one participant per study arm and a $20 gift card for 1 participant per team at 12 weeks, as well as a drawing for 1 $200 gift card, 4 $100 gift cards, and 5 t-shirts valued at $25 across both arms, and $25 gift cards for up to 2 participants per team at 52 weeks). The study was approved by the Institutional Review Board at the University of South Carolina.

### Participants

Individuals were eligible if they were between ages 18 and 65 years, were insufficiently physically active (i.e., had an average daily baseline step count of < 7500 [1, 74, 75] measured via ActiGraph accelerometry over one week), a body mass index (BMI; kg/m^2^) between 18 and 55, access to the internet, owned a smartphone (iPhone or Android), and were part of a self-selected team of 2 to 7 other eligible persons. Individuals were ineligible if they were pregnant, lactating, or planning to become pregnant within 1 year of enrollment, had diabetes or a medical contraindication for engaging in moderate-intensity PA, or enrolled in another PA program. Members of the same household were eligible to participate if they were on the same team.

### Randomization

Teams were randomly assigned to either a 12-week standard technology-delivered PA intervention (TECH) or the same intervention plus a step competition and PA challenge game (TECH + Gamification) by the lead statistician, using a computer-based random number generator in a 1:1 ratio, stratified by team size.

### Intervention

#### Common intervention components

Each study arm’s PA intervention was rooted in the social cognitive theory (SCT) [76]. Participants were prescribed a step goal (average of ≥ 1,000 steps/d during week 1, ≥ 2,000 steps/d during week 2, and ≥ 3,000 steps/d each week thereafter above their personal average daily baseline step count). This daily step goal reflects an incremental progression towards 3,000 steps/d which approximates the recommended PA guidelines [75]. They received and got to keep a wrist-worn Fitbit Alta HR PA tracker, which continuously displays steps and active minutes among other metrics. It wirelessly syncs with the Fitbit app, allowing participants to track their PA progress and interact with other Fitbit users. Participants were also asked to access a secure, password-protected, responsive-design study website to view weekly behavior change content focused on goal setting, self-monitoring, planning, social support, problem solving, and relapse prevention. Each participant’s Fitbit data were accessible via the Fitbit application programing interface and automatically drawn into the study website, allowing for graphical displays of their personal individual daily step count progress over time, each of their teammate’s contribution to their team’s daily step count progress over time, and their team’s collective average daily step count progress over time relative to all other teams combined in their study arm. Based on these data, four experts with an advanced degree and corresponding experience in exercise science, public health, and/or behavioral psychology served a specific interventionist role, sending participants a personalized weekly electronic feedback message about their step goal progress during the first intervention month that followed a standard suggested framework. The website also contained a study blog curated by one of these interventionists that supplemented the weekly behavior change content, as well as a journal feature where participants could record messages of self-reflection.

#### *TECH* + Gamification

The TECH + Gamification group also engaged with custom-designed gamification elements underpinned by the SCT [76], self-determination theory [77], social network theory [76], and behavioral economics principles [73, 76, 78–80]. Teams participated in a step competition delivered through the study website. Each team’s cumulative average step count was updated and displayed on a leaderboard in near real-time based on their incoming Fitbit data, along with corresponding team rankings. Participants were required to sync their data to the Fitbit app at a minimum by the end of each week for it to count towards the competition. The team with the most average cumulative steps by the end of the 12-week intervention won the step competition.

Teams also participated in a weekly PA challenge game called the Shoe Mascot Game, which was supported by the incoming Fitbit data. Each team had a virtual shoe mascot avatar. The object of the game was to keep their shoe mascot on a virtual walking trail (highest level of the game) and away from three lower levels of the game represented by sedentary objects by achieving two weekly PA challenges that were presented at the start of each week on the study website. Each PA challenge had a corresponding point value. Some challenges were team-based (e.g., your team must collectively accumulate more steps this week versus the prior week), and others were more individual oriented (e.g., one team member will be randomly selected to see if they achieved their personal step goal for the week). During some weeks, teams could pick their challenge from options. If the challenges were met, then the team would not lose points, and their shoe mascot stayed on the walking trail. If the challenges were not fulfilled, then the team would lose points and fall to lower levels, with only one chance to return to the walking trail by achieving a bonus challenge. Team rankings for the game were displayed on the leaderboard, and the team that retained the most points by the end of the 12-week intervention won the game. Winning teams of the step competition and Shoe Mascot Game received a congratulatory message on the website that was visible to all other teams.

### Outcome measures

#### Treatment adherence

Mean total number of days per week that participants self-monitored their PA, mean proportion of participants who self-monitored PA at least one day per week, and mean proportion who self-monitored PA all 7 days per week using their Fitbit, were calculated for both the 12-week intervention period and 40-week post-intervention period. An accumulated step count of 500 or more on any given day represented a valid day of self-monitoring [81–83]. Log-ins to the study website were also monitored during the 12-week intervention.

#### Treatment satisfaction

At the end of the intervention, participants were asked via online questions about their satisfaction with the program and willingness to recommend it to family, friends, or co-workers using a 5-point Likert-type scale, with lower scores reflecting strong dissatisfaction and unlikeliness to recommend it and vice versa.

#### ActiGraph accelerometer-measured steps and MVPA

Daily steps and MVPA minutes were measured using the ActiGraph at baseline, 12 weeks, and 52 weeks. Participants were instructed to wear the device during all waking hours (except when showering or swimming) for 7 consecutive days on their non-dominant hip using a provided waistband and pouch. Data were sampled at a frequency of 90 Hz. Using ActiLife 6 software (version 6.13.3), raw accelerometer data were processed into 60-s epochs and subsequently scored using the Troiano 2008 adult cut points for classifying MVPA [84], with periods of non-wear time (≥ 90 min of continuous zeroes) excluded from analysis. Data were considered valid if the device was worn for at least 3 days [85–87] for at least 10 h/d [88] and subsequently mean values for steps/d and MVPA min/d were calculated for each of the three respective weekly measurement periods.

#### Fitbit-measured steps and active minutes

Data were considered valid if an accumulated daily step count of 500 or more was achieved on 3 or more days for a given week [81–83, 85, 86, 89]. Mean daily steps and active minutes (i.e., sum of fairly active and very active minutes, reflecting a PA intensity of 3 or more metabolic equivalents, and thus, MVPA) values derived from the Fitbit were calculated across the 12-week intervention period and 40-week post-intervention period.

#### Social support for exercise

Perceived social support from family and friends combined for exercise (SSE) was measured online using the valid and reliable 13-item Sallis Social Support Scale for Exercise [90] at baseline, 12 weeks, and 52 weeks. Participants rated each item on a 5-point scale. Item scores were averaged, with higher scores indicating a stronger perception of support.

#### Sociodemographic characteristics

Sociodemographic characteristics were reported at baseline using an online questionnaire.

#### Body weight and height

Weight was measured at baseline to the nearest 0.1 kg in street clothes, without shoes, using a calibrated digital scale (Tanita BWB 800, Arlington Heights, IL). Height was measured at baseline to the nearest 0.1 cm using a standard stadiometer. BMI was calculated as weight (kg) per height (m^2^).

### Statistical analyses

Descriptive statistics were calculated for baseline demographic measures, retention rates, and all outcomes. The generalized linear model with logit link was used for investigating the predictors for non-completers at 12 weeks and 52 weeks. Primary outcomes were treatment adherence, study retention, treatment satisfaction, and change in ActiGraph-measured daily steps from baseline to 12 weeks. Changes in ActiGraph-measured daily steps from baseline to 52 weeks, ActiGraph-measured daily MVPA minutes from baseline to 12 weeks and baseline to 52 weeks, SSE from baseline to 12 weeks and baseline to 52 weeks, and Fitbit-measured daily steps and active minutes aggregated across the 12-week intervention period and 40-week post-intervention period were secondary outcomes.

For the missing ActiGraph accelerometer data, the generalized estimating equation model (GEE) [91] was fit to study the association between the non-completers and all variables with adjustment of repeated measures among participants and the teams. It showed that there was no significant association, indicating the missing completely at random (MCAR) assumption was appropriate [91]. Then, the GEE was applied for each outcome of interest with adjustment of repeated measures among participants and the teams. The main model was estimated by also adjusting for sex, age, race/ethnicity, education, marital status, BMI, team size, and wear time for each outcome of interest. Based on the marginal models, differences in the changes from baseline to week 12 and from baseline to week 52 for mean daily steps and MVPA minutes were investigated between the two groups.

For the SSE data, the missing data were not in a monotonic pattern and thus assumed to be missing at random (MAR). Multiple imputations were conducted for the missing values based on the Markov Chain Monte Carlo method. The final results from the imputed data were based on 10 imputed data sets [92]. The main mixed effects model was applied for the outcome with adjustment of repeated measures among participants and the teams. The main model was estimated by also adjusting for sex, age, race/ethnicity, education, marital status, BMI, and team size for the outcomes of interest. Based on the mixed effects models, the differences of the change in SSE from baseline to week 12 and from baseline to week 52 were investigated between the two groups.

Missing Fitbit step data and corresponding active minutes data were not in a monotonic pattern and thus assumed to be missing at random (MAR), and the aforementioned methods were applied. Two-sample *t*-tests were used to measure differences between groups for average number of Fitbit-measured daily steps, active minutes, and days of self-monitoring aggregated across the 12-week intervention period and the 40-week post-intervention period. Chi-squared tests were used to measure differences between groups in the proportions of participants who self-monitored at least one day per week, as well as the proportions of participants who self-monitored all 7 days per week aggregated across the 12-week intervention period and the 40-week post-intervention period. The mixed effects models were applied for each outcome of interest with adjustment of sex, age, race/ethnicity, education, marital status, BMI, team size, and teams.

A two-sample *t*-test and chi-squared test were used to measure the differences between groups for mean number of log-ins and the proportion of participants who logged in at least one time per week, respectively, aggregated across the 12-week intervention period. The mixed effects models were applied for each outcome of interest with adjustment of sex, age, race/ethnicity, education, marital status, BMI, team size, and teams. Statistical significance was set at 0.05. SAS 9.4 was used for all analyses.

## Results

**Fig. 1:**
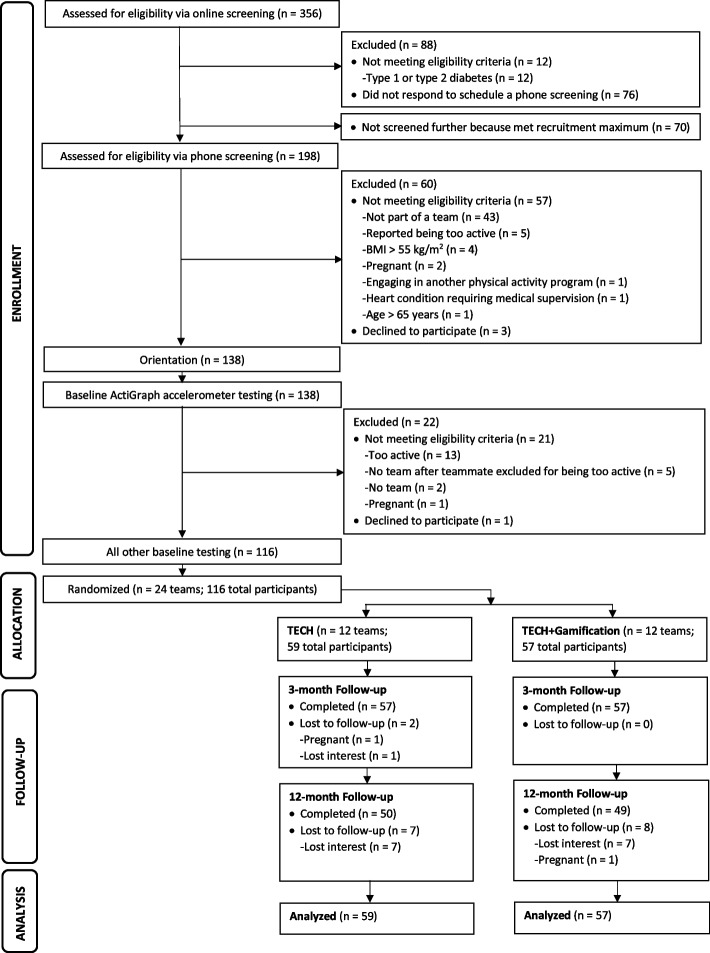
CONSORT flow diagram

The flow of participants through the study is shown in Fig. 1 (See Additional File 1 for CONSORT checklist). Twenty-four teams (*N* = 116 total participants) were randomized to one of two conditions. Participants were mostly female (78%), middle aged, White, and well educated. At 12 weeks and 52 weeks, 98% and 85% of participants were retained, respectively (Table 1). No sociodemographic characteristics were predictors of participants being lost to follow-up at 12 weeks. At 52 weeks, participants without a college degree had 9.5 times (95% CI, 2.0 to 46.1) greater odds of dropping out versus those with a college degree. ActiGraph wear compliance among those who engaged in the measurement was high at each time point (See Additional File 2). During the 12-week intervention period, Fitbit data that were missing, or that had step values of less than 500 steps per day, represented 6.09% and 2.26% of observations in TECH and TECH + Gamification, respectively. During the 40-week post-intervention period, these percentages increased to 43.42% and 30.67%, respectively (See Additional File 3).

**Table 1:** Baseline characteristics and retention rates

Measure	All (*N* = 116)	TECH + Gamification (*N* = 57)	TECH (*N* = 59)
Age, years	40.14 (10.31)	40.60 (11.93)	39.69 (8.55)
Female, % (*f*)	78.45 (91)	78.95 (45)	77.97 (46)
Race, % (*f*)			
White	66.38 (77)	64.91 (37)	67.80 (40)
African American	25.86 (30)	22.81 (13)	28.81 (17)
Asian	4.31 (5)	8.77 (5)	0.00 (0)
Mixed race	3.45 (4)	3.51 (2)	3.39 (2)
Education, %( *f*)			
Bachelor’s degree or higher	79.31 (92)	68.42 (39)	89.83 (53)
Relationship Status, % (*f*)			
Married	62.93 (73)	61.40 (35)	64.41 (38)
Living as married	1.72 (2)	1.75 (1)	1.69 (1)
Divorced	6.90 (8)	5.26 (3)	8.47 (5)
Separated	1.72 (2)	3.51 (2)	0.00 (0)
Widowed	0.86 (1)	1.75 (1)	0.00 (0)
Single	25.86 (30)	26.32 (15)	25.42 (15)
BMI, kg/m^2^	33.05 (6.85)	32.03 (6.58)	34.03 (7.02)
Team Size	5.24 (1.54)	5.28 (1.81)	5.20 (1.23)
MVPA, min/d^a,b^	15.05 (9.39)	14.90 (9.18)	15.19 (9.66)
Steps/d^b^	4853 (1333)	4990 (1286)	4721 (1375)
Wear Time, min/d^c^	855.04 (69.48)	867.15 (80.52)	843.33 (55.03)
Valid Days^d^	6.43 (0.84)	6.60 (0.60)	6.27 (1.0)
Social Support for Exercise^e^	2.48 (0.55)	2.62 (0.58)	2.34 (0.49)
Retained for 12-week follow-up, % (*f*)	98.28 (114)	100.0 (57)	96.61 (57)
Retained for 52-week follow-up, % (*f*)	85.34 (99)	85.96 (49)	84.75 (50)

Data are mean (SD) unless indicated by % (*f*)

^a^moderate-to-vigorous PA

^b^as measured by ActiGraph accelerometer

^c^time spent wearing ActiGraph accelerometer

^d^number of valid days of ActiGraph accelerometer wear

^e^Score range 1 to 5, with 5 indicating high perceived support

### Treatment adherence

Based on the two-sample *t*-tests, significant differences were found between groups in the mean number of days per week of self-monitoring PA using the Fitbit during the 12-week intervention period (TECH, 6.58 ± 1.23; TECH + Gamification, 6.86 ± 0.63; *P* < 0.01) and the 40-week post-intervention period (TECH, 4.01 ± 3.21; TECH + Gamification, 4.85 ± 2.93; *P* < 0.01). In the adjusted mixed effects models, the differences in self-monitoring rates were significant during the intervention period (-0.30; 95% CI, -0.54 to -0.07; *P* = 0.01) but not during the post-intervention period (-1.22; 95% CI, -2.49 to 0.04; *P* = 0.06).

**Fig. 2:**
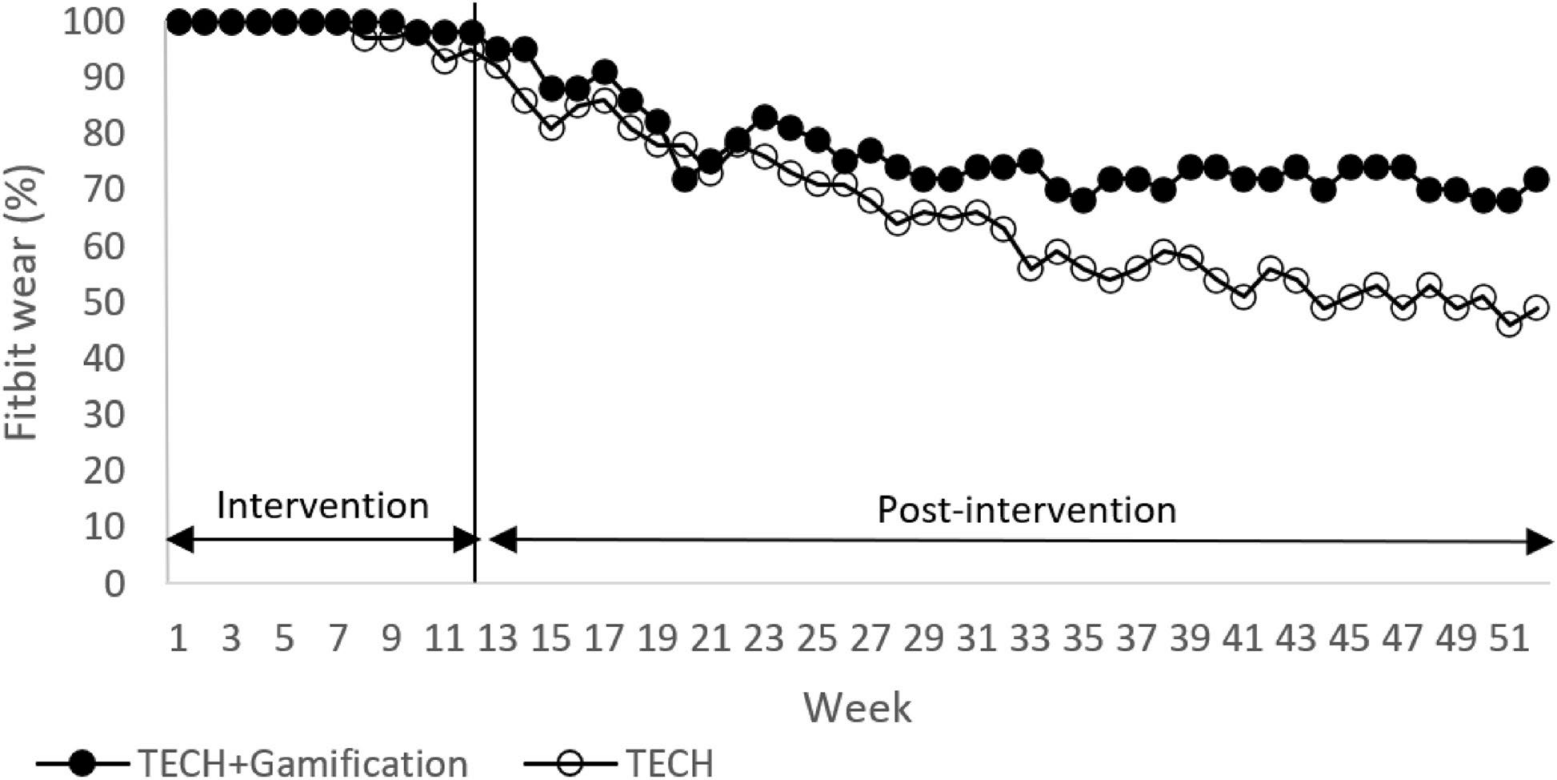
Percentage of Fitbit wear indicates the percentage of participants who wore the Fitbit for at least 1 day in that week. Any day during which a participant logged 500 or more steps on the Fitbit was regarded as a valid day and constituted wearing the tracker for that day

Figure 2 shows the percentage of participants in each group who self-monitored their PA at least one day per week using the Fitbit over time. Based on the chi-squared test, on average, a significantly lower proportion of TECH participants self-monitored their PA at least one day per week vs TECH + Gamification across the 12-week intervention period (98% vs 99%; *P* = 0.02), but in the adjusted mixed effects model, the observed odds ratio predicting self-monitoring of PA at least one day per week over the 12-week intervention period was not significant (0.40, 95% CI, 0.05 to 3.28, *P* = 0.39). A lower mean proportion of TECH participants self-monitored their PA at least one day per week across the 40-week post-intervention period versus TECH + Gamification based on the chi-squared test (64% vs 76%, *P* < 0.01), and in the adjusted mixed effects model, TECH had 63% lower odds vs TECH + Gamification (0.37, 95% CI, 0.18 to 0.77, *P* = 0.01) of self-monitoring their PA at least one day per week.

**Fig. 3:**
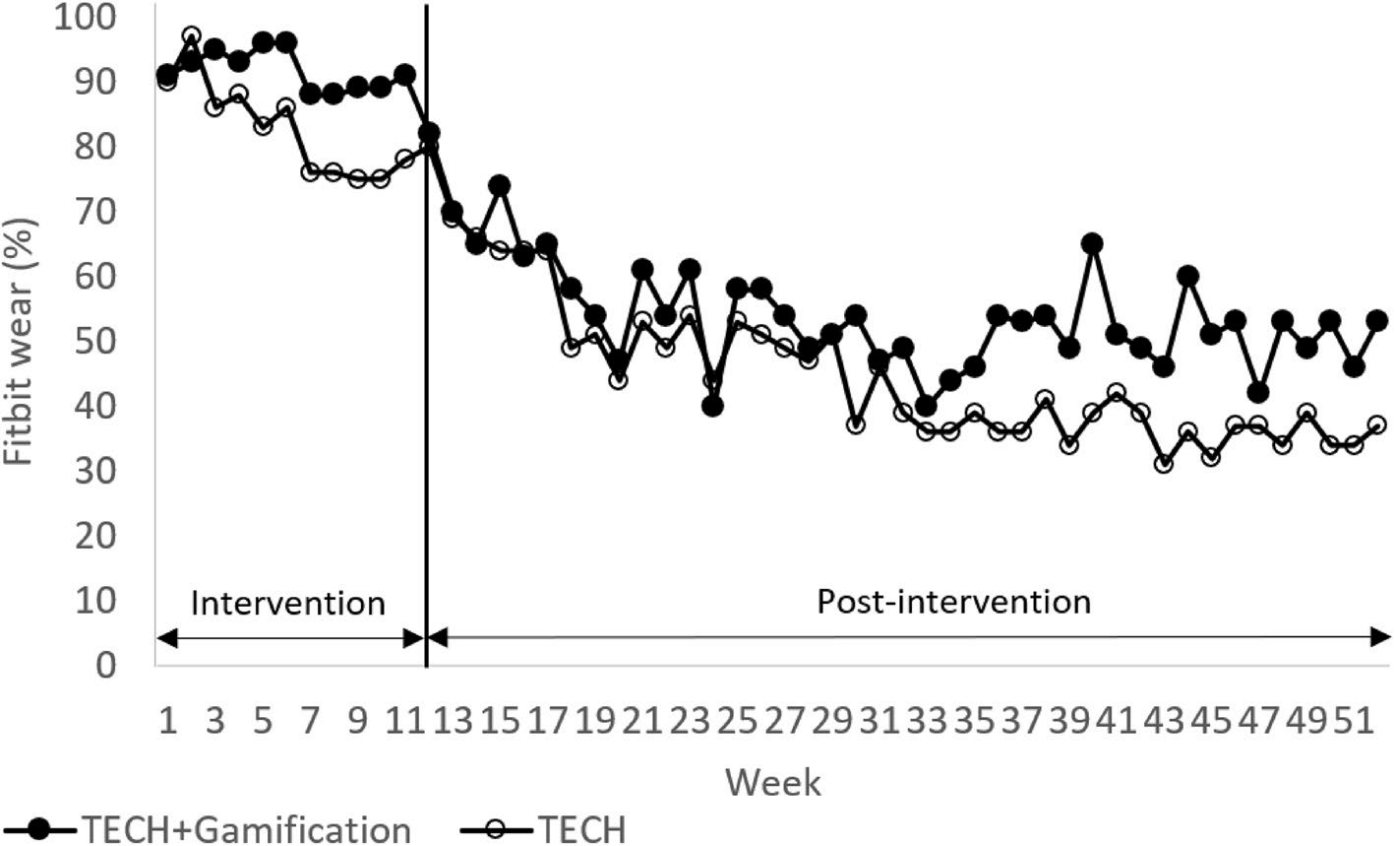
Percentage of Fitbit wear indicates the percentage of participants who wore the Fitbit all 7 days in that week. Any day during which a participant logged 500 or more steps on the Fitbit was regarded as a valid day and constituted wearing the tracker for that day

Figure 3 shows the percentage of participants in each group who self-monitored their PA all 7 days per week using the Fitbit over time. Based on the chi-squared test, on average, a significantly lower proportion of TECH participants self-monitored their PA all 7 days per week vs TECH + Gamification across the 12-week intervention period (82% vs 91%; *P* < 0.01), but, in the adjusted mixed effects model, the observed odds ratio regarding self-monitoring of PA was not significant (0.56; 95% CI, 0.32 to 1.00; *P* = 0.05). A lower mean proportion of TECH participants self-monitored their PA all 7 days per week across the 40-week post-intervention period versus TECH + Gamification based on the chi-squared test (44% vs 54%, *P* < 0.01), and in the adjusted mixed effects model, TECH had 47% lower odds vs TECH + Gamification (0.53; 95% CI, 0.31 to 0.89; *P* = 0.02) of self-monitoring their PA all 7 days per week.

Based on the two-sample *t*-test, a significant difference was found between groups in the mean number of log-ins per week during the 12-week intervention period (TECH, 1.18 ± 3.65; TECH + Gamification, 8.47 ± 15.75; *P* < 0.01). In the adjusted mixed effects model, the difference in log-in rates remained significant during the intervention period (-7.14; 95% CI, -10.60 to -3.67; *P* < 0.01).

**Fig. 4:**
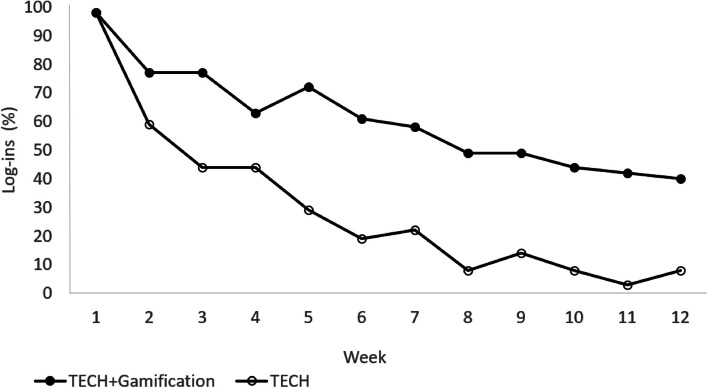
Percentage of log-ins indicates the percentage of participants who logged in to the website at least 1 time in that week during the 12-week intervention

Figure 4 shows the percentage of participants in each group who logged in at least one time per week over the 12-week intervention period. Based on the chi-squared test, on average, a significantly lower proportion of TECH participants logged in at least one time per week vs TECH + Gamification across the 12-week intervention period (30% vs 61%; *P* < 0.01), and in the adjusted mixed effects model, TECH had 75% lower odds vs TECH + Gamification (0.25; 95% CI, 0.14 to 0.42; *P* < 0.01) of logging in at least one time per week.

### Treatment satisfaction

Most TECH + Gamification (96%) and TECH (92%) participants agreed or strongly agreed that they would recommend the program to a friend, family member, or co-worker, and 91% and 90% indicated they were satisfied with their respective program.

### ActiGraph accelerometer-based PA changes

The unadjusted mean (SD) change in steps per day from baseline to post-treatment (12 weeks) was 939 ± 2068 for TECH versus 532 ± 1373 for TECH + Gamification. At 52 weeks, the unadjusted mean (SD) change in steps per day was 577 ± 2175 for TECH versus 231 ± 1758 for TECH + Gamification. In the model there were no significant differences between the two groups in changes in steps per day from baseline to post-treatment (12 weeks) (-425; 95% CI, -1065 to 215; *P* = 0.19) and from baseline to 52 weeks follow-up (-277; 95% CI, -1043 to 489; *P* = 0.48). Analyses that adjusted for covariates demonstrated similar results (Table 2).

**Table 2:** ActiGraph accelerometer PA outcomes

	Mean (SD)		Model^f^		Model adjusted for covariates^g^	
Measure	TECH	TECH + Gamification	Gamification Effect^h^	*P* Value	Gamification Effect^h^	*P* Value
Baseline						
Steps/d	4721 (1375)^b^	4990 (1286)^c^	NA	NA	NA	NA
MVPA, min/d^a^	15.19 (9.66)^b^	14.90 (9.18)^c^	NA	NA	NA	NA
12 weeks						
Steps per day	5695 (2527)^c^	5522 (1980)^c^	-425 (-1065–215)	.19	-274 (-882–334)	.38
MVPA, min/d^a^	21.56 (18.54)^c^	17.78 (12.75)^c^	-3.36 (-8.62–1.91)	.21	-2.66 (-7.88–2.55)	.31
Follow-up, 52 weeks						
Steps/d	5339 (2156)^d^	5361 (2099)^e^	-277 (-1043–489)	.48	-133 (-868–601)	.72
MVPA, min/d^a^	19.19 (17.25)^d^	16.94 (14.57)^e^	-1.67 (-7.84–4.50)	.60	-0.95 (-6.92–5.02)	.75

^a^moderate-to-vigorous PA

^b^*N* = 59

^c^*N* = 57

^d^*N* = 50

^e^*N* = 49

^f^Adjusted for repeated measures and team random effect

^g^Adjusted for repeated measures, team random effect, age, BMI, marital status, sex, race/ethnicity, education, team size, and wear time

^h^The TECH + Gamification arm is compared with the TECH arm during the specified periods

The unadjusted mean (SD) change in minutes per day of MVPA from baseline to post-treatment (12 weeks) was 6.02 ± 17.59 for TECH versus 2.88 ± 10.42 for TECH + Gamification. At 52 weeks from baseline, the unadjusted mean (SD) change in minutes per day of MVPA was 3.46 ± 18.52 for TECH versus 2.08 ± 13.20 for TECH + Gamification. In the model (Table 2) there were no significant differences between the two groups in changes in minutes per day of MVPA from baseline to post-treatment (12 weeks) (-3.36; 95% CI, -8.62 to 1.91; *P* = 0.21) and from baseline to 52 weeks follow-up (-1.67; 95% CI, -7.84 to 4.50; *P* = 0.60). Analyses that adjusted for covariates showed similar results (Table 2).

### Fitbit-based PA outcomes

**Fig. 5:**
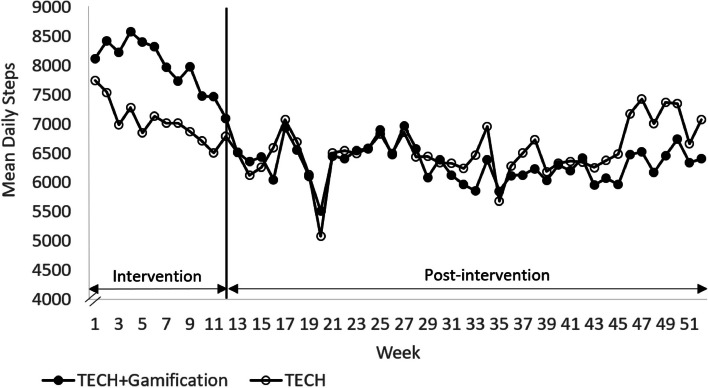
Fitbit derived mean number of daily steps

Figure 5 shows the mean number of steps in each group over time. Based on the two-sample *t*-test, a significant difference was found between groups in the mean number of total daily steps over the entire 12-week intervention period (TECH, 7041 ± 2520; TECH + Gamification, 7988 ± 2707; *P* = 0.02), but in the adjusted model, the difference was not significant (-1165; 95% CI, -2443 to 114; *P* = 0.07). No significant difference between groups was found in the mean number of total daily steps over the entire 40-week post-intervention period (TECH, 6542 ± 2710; TECH + Gamification, 6317 ± 2457; *P* = 0.45) based on the two-sample *t*-test, and the difference remained non-significant in the adjusted model (125; 95% CI, -604 to 854; *P* = 0.74).

**Fig. 6:**
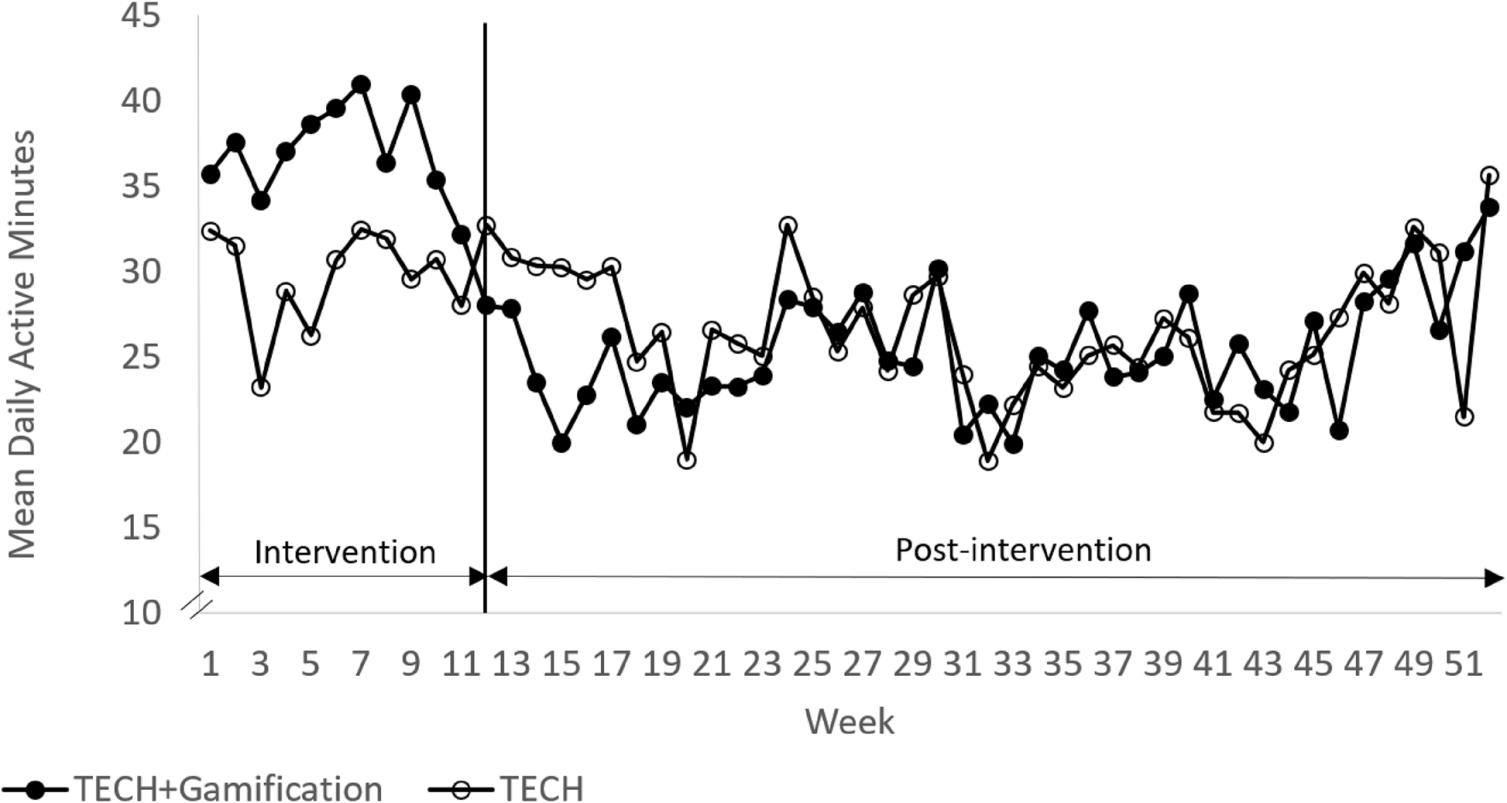
Fitbit derived mean number of daily active minutes

Figure 6 shows the mean number of active minutes in each group over time. Based on the two-sample *t*-tests, no significant differences were found in the mean number of total daily active minutes between conditions both over the 12-week intervention period (TECH, 29.90 ± 29.76; TECH + Gamification, 36.38 ± 29.83; *P* = 0.14) and 40-week post-intervention period (TECH, 26.50 ± 32.40; TECH + Gamification, 25.26 ± 33.19; *P* = 0.74). Similar results were observed in the adjusted models (-4.68; 95% CI, -14.92 to 5.56;* P* = 0.37, and 2.34; 95% CI, -6.77 to 11.45;* P* = 0.62, respectively).

### Social support changes

The unadjusted mean (SD) change in SSE from baseline to post-treatment (12 weeks) was 0.46 ± 0.70 for TECH versus 0.50 ± 0.67 for TECH + Gamification. At 52 weeks from baseline, the unadjusted mean (SD) change in SSE was 0.41 ± 0.59 for TECH versus 0.41 ± 0.59 for TECH + Gamification. In the model there was no significant difference between the two groups in SSE from baseline to post-treatment (12 weeks) (0.04; 95% CI, -0.21 to 0.29; *P* = 0.76) and from baseline to 52 weeks follow-up (-0.02; 95% CI, -0.25 to 0.21; *P* = 0.87). Analyses that adjusted for covariates demonstrated similar results (Table 3).

**Table 3:** Social support outcomes

	Mean (SD)		Model^e^		Model adjusted for covariates^f^	
Measure	TECH	TECH + Gamification	Gamification Effect^g^	*P* Value	Gamification Effect^g^	*P* Value
Baseline	2.34 (0.49)^a^	2.62 (0.58)^b^	NA	NA	NA	NA
12 weeks	2.80 (0.65)^b^	3.12 (0.64)^b^	0.04 (-0.21 to 0.28)	.76	0.04 (-0.21 to 0.28)	.76
Follow-up, 52 weeks	2.76 (0.60)^c^	3.01 (0.66)^d^	-0.02 (-0.25 to 0.21)	.87	-0.02 (-0.25 to 0.21)	.87

^a^*N* = 59

^b^*N* = 57

^c^*N* = 49

^d^*N* = 48

^e^Adjusted for repeated measures and team random effect

^f^Adjusted for repeated measures, team random effect, age, BMI, marital status, sex, race/ethnicity, education, and team size

^g^The TECH + Gamification arm is compared with the TECH arm during the specified periods

## Discussion

This study assessed the feasibility and preliminary efficacy of a socialincentive-based gamification approach for improving PA. The findings suggest that the addition of a step competition and weekly PA challenge game to an e/mHealth PA intervention among teams of insufficiently active adults with existing social ties is feasible and acceptable. While in place, this gamification intervention facilitated positive, clinically meaningful differences in Fitbit-measured daily steps and active minutes versus an identical program without gamification. However, once withdrawn, it did not result in increases in ActiGraph-measured daily steps, MVPA, and social support over and above what was observed with the traditional e/mHealth treatment alone.

This is the first e/mHealth gamification PA study among teams of adults with existing social ties to measure participants’ PA using both research grade and commercial accelerometers over one year via an RCT, yielding valuable insights about both the short- and long-term effects of team-based gamification among adults. Metrics regarding adherence to self-monitoring PA using the Fitbit were high for both study arms during the intervention but slightly declined thereafter. A more favorable, self-monitoring profile was observed for TECH + Gamification versus TECH, suggesting that the gamified elements had the desired effect of creating a heightened culture of accountability [93] by prompting participants to frequently track and sync their PA to ensure their team got credit for it. These findings are consistent with those from a previous micro-randomized trial among adults in which a positive causal effect of a smartphone-based gamified team competition was found for the proportion of days that participants on the team provided daily steps [72]. In the current study, nearly three-quarters of TECH + Gamification participants were still self-monitoring their PA at least one day per week versus half for TECH by the end of the 40-week post-intervention period. It is possible that the self-monitoring behaviors established while each respective intervention was in place turned into habits that persisted, but in the absence of the other theory-based gamification elements, it was potentially not impactful enough to sustain the PA levels observed during the intervention. Interestingly, in a 6-month, financial incentive-based PA promotion RCT targeting individual employees, only 10% of participants across treatment conditions were self-monitoring PA using their Fitbit at one-year based on the same valid day criterion used in the current study [81]. The higher adherence to self-monitoring PA across both arms in the present study at one year might be due to leveraging teams of existing social ties instead of individuals, potentially creating a social climate that increased the likelihood of sustained self-monitoring. Future team-based PA promotion studies should examine social processes among teammates and their relationship to self-monitoring and PA behaviors.

Consistent with prior studies, [58, 94–96] gamification resulted in greater engagement with the study web app as reflected by log-in rates during the intervention, plausibly because participants were keeping abreast of the team competition and weekly challenge game. Although not statistically different, a similar pattern was observed for Fitbit-measured daily steps and active minutes during the intervention, with an average difference of 947 steps and just over 6 active minutes favoring TECH + Gamification versus TECH. These represent clinically meaningful, health-promoting PA values that could have an impactful effect at the population level [2, 97] and are situated among mixed short-term findings from previous gamification PA RCTs using device-based measures among adults [37, 49–55, 66, 68, 69, 71, 72, 98–102]. Taken together, it is possible that the overall enhanced intervention adherence effects of gamification largely drove these differences in Fitbit-measured physical activity during the intervention via more frequent exposure to theory-based content on the study web app, meaningful social interactions around physical activity, and enactment of self-regulation strategies (self-monitoring progress towards goals which is a strong predictor of physical activity behavior change) [9].

The game-based strategies did not confer an added benefit in Fitbit-measured PA during the post-intervention period. Relatedly, similar, modest increases in ActiGraph-measured daily steps and MVPA were captured immediately after intervention removal in both study arms, and these improvements slightly declined to a similar degree; however, they remained above baseline by 1 year, perhaps due to factors related to being part of a team [42, 103]. The intent of targeting teams of individuals with existing social ties was to harness a social structure conducive to sustaining support for PA – a key contributor to maintaining regular PA participation [20] – potentially ignited by the gamification elements. However, similar increases in social support were observed in both study arms immediately post-intervention, and these improvements drifted back towards baseline by 1 year. These findings indicate that engaging teams of insufficiently active adults with existing social ties in a gamified, e/mHealth PA program is not sufficient to elevate social support to an extent that drives sustained PA enhancements beyond a program without these gamification elements. While the implemented gamification approach did not drive lasting, enhanced PA changes, it does not necessarily indicate that existing social ties are not potent or influential; rather, there may be better ways of leveraging the support inherent within them. For instance, directly gamifying social support and/or providing formal training on how teammates can best support each other [104, 39] may be needed to facilitate desired long-term outcomes and should be explored in future investigations.

It is possible that the gamified approach served largely as an extrinsically oriented motivating factor that had a positive effect while in place but inability to drive sustained PA participation once removed. This factor in combination with the lack of an enhanced gamification effect on social support may have stifled the chance to develop intrinsic motivation which is a strong predictor of PA adherence [105–107]. Similarly, a previous PA promotion RCT examining the effectiveness of Fitbit trackers and financial incentives observed an undermining effect of cash incentives, finding short-term increases in daily steps that regressed over a 6-month follow-up period upon incentive removal [81]. A recent meta-analysis of evidence regarding the effectiveness of gamification on PA [63] found a small but weak long-term (12 to 24 weeks follow-up) effect for these interventions. Future PA promotion research should experimentally seek to determine the optimal integration of social incentive-based gamified elements with individual, relational, and team characteristics (e.g., race/ethnicity, sex, employment, education, starting PA level, geographic location, type of relationships, team size, etc.) for promoting regular PA. Using formative research to inform intervention design [107] and ecological momentary assessment to capture frequent participant perspectives during and after treatment could yield valuable insights in this realm, including the psychosocial mechanisms underlying observed effects.

Further, future research should explore whether novel “booster” gamification doses (e.g., additional challenges) should be implemented during a maintenance phase [57, 59, 63, 64]. Previous studies have shown that novelty assists with reinforcement learning and reward processing [108]. For example, a population-wide mHealth intervention in Singapore implemented individual- and corporate team-based booster step challenges along with small prizes following the main intervention period which resulted in additional increases in steps [109]. In the U.S., previous employer-sponsored and statewide, team-based physical activity campaigns have demonstrated promise for increasing physical activity [41, 110, 111]. Going forward, understanding how best to maximize the long-term impact of e/mHealth gamification approaches involving social networks, ongoing booster initiatives, and small incentives on a large scale via delivery across entities with widespread reach (e.g., commercial, corporate, statewide, etc.) should be a priority.

In the current study, it was not possible to disentangle the effects of the team competition and weekly challenge game on the outcomes, which reflects one limitation. Another limitation is the predominantly female and highly educated sample, reducing the generalizability of the findings. However, this study has several strengths, including the following: 40-week post-intervention period; two device-based PA measures; use of an RCT to isolate the efficacy of a custom gamification approach delivered through technologies, enhancing scalability potential; measurement of social support; high retention; and recruitment of teams with varying combinations of existing social ties (friends; family; co-workers).

## Conclusions

Augmenting a theory-based e/mHealth PA promotion program with gamification elements designed to tap into social support within existing social ties was feasible and resulted in a positive, clinically meaningful additive PA effect while in place but did not enhance PA once withdrawn. Given the support inherent within existing social ties, increasing ubiquitousness of health-promoting technologies, and heightened commercial and scientific interest in gamification, continued exploration of methods that capitalize on the combination of these three factors for affecting PA and overall well-being is warranted.

### Supplementary Information

**Additional file 1. **CONSORT checklist.

**Additional file 2. **ActiGraph accelerometer wear time and number of valid days of accelerometer weara.

**Additional file 3. **Missing Fitbit data by study arm and period.

**Additional file 4. **The TIDieR (Template for Intervention Description and Replication) Checklist*: Information to include when describing an intervention and the location of the information.

**Additional file 5. **Sample and missing data description.

## Data Availability

The data sets analyzed during the current study are available from the corresponding author upon reasonable request.

## Abbreviations

PA: Physical activity
e/mHealth: Electronic and mobile health
MVPA: Moderate to vigorous physical activity
RCT: Randomized controlled trial
SCT: Social cognitive theory
BMI: Body mass index
GEE: Generalized estimating equation model
MAR: Missing at random
MCAR: Missing completely at random
SSE: Social support for exercise
