# Supplementary material for: Harnessing technology and gamification to increase adult physical activity: a cluster randomized controlled trial of the Columbia Moves pilot
Source: Int J Behav Nutr Phys Act. 2023 Nov 3;20:129. doi: 10.1186/s12966-023-01530-1 (PMC10623775; doi:10.1186/s12966-023-01530-1)
Supplement: Supplementary file 2 — Additional file 2. ActiGraph accelerometer wear time and number of valid days of accelerometer weara. [file 12966_2023_1530_MOESM2_ESM.pdf]

Additional File 2. ActiGraph accelerometer wear time and number of valid days of accelerometer wear[a]

Time	Study Arm	n[b]	Mean (SD) wear time, min	3 days, %	4 days, %	5 days, %	6 days, %	7 Days, %
Baseline	TECH	59	843.33 (55.03)	1.69	5.08	13.56	23.23	55.93
	TECH+Gamification	57	867.15 (80.52)	0.00	0.00	5.26	29.82	64.91
12 weeks	TECH	57	871.09 (130.87)	0.00	21.05	15.79	19.30	43.86
	TECH+Gamification	57	862.85 (74.44)	0.00	19.30	8.77	22.8	49.12
Follow-up, 52 weeks	TECH	50	883.27 (150.08)	8.00	20.00	12.00	22.00	38.00
	TECH+Gamification	49	878.83 (109.56)	12.24	10.20	10.20	22.45	44.90

[a]A valid day was defined as having 10 or more hours of accelerometer wear

[b]Reflective of study completers (all who engaged in the measurement met the wear time criterion of 3 or more valid days during the week)
