# Supplementary material for: Harnessing technology and gamification to increase adult physical activity: a cluster randomized controlled trial of the Columbia Moves pilot
Source: Int J Behav Nutr Phys Act. 2023 Nov 3;20:129. doi: 10.1186/s12966-023-01530-1 (PMC10623775; doi:10.1186/s12966-023-01530-1)
Supplement: Supplementary file 3 — Additional file 3. Missing Fitbit data by study arm and period. [file 12966_2023_1530_MOESM3_ESM.pdf]

Additional File 3. Missing Fitbit data by study arm and period

Variable	TECH (n=59)	TECH+Gamification (n=57)
PA (steps per day)		
Weeks 1-12		
Value of “0” either because did not self-monitor or sync data	241/4956 participant-days (4.86%)	78/4788 participant-days (1.63%)
Value 1 to 499	61/4956 participant-days (1.23%)	30/4788 participant-days (0.63%)
Weeks 13-52		
Value of “0” either because did not self-monitor or sync data	6736/16520 participant-days (41.45%)	4591/15960 participant-days (28.77%)
Value 1 to 499	320/16520 participant-days (1.97%)	303/15960 participant-days (1.90%)
