# Supplementary material for: Harnessing technology and gamification to increase adult physical activity: a cluster randomized controlled trial of the Columbia Moves pilot
Source: Int J Behav Nutr Phys Act. 2023 Nov 3;20:129. doi: 10.1186/s12966-023-01530-1 (PMC10623775; doi:10.1186/s12966-023-01530-1)
Supplement: Supplementary file 5 — Additional file 5. Sample and missing data description. [file 12966_2023_1530_MOESM5_ESM.pdf]

Additional File 5. Sample and missing data description

A convenience sample of participants (78% female; 66% White; 79% bachelor's degree or higher; 63% married) was recruited from the Greater Columbia, SC area via flyers, e-mails sent via listservs, and word of mouth between January 2018 and April 2018. According to the U.S. Census Bureau, Richland County, South Carolina (Greater Columbia area), is 52% female and 45% White, with 40% of persons holding a bachelor's degree or higher and 43% who are married. For the missing ActiGraph accelerometer data, the generalized estimating equation model was fit to study the association between the non-completers and all variables with adjustment of repeated measures among participants and the teams. It showed that there was no significant association, indicating the missing completely at random assumption was appropriate. For the social support for exercise data, the missing data were not in a monotonic pattern and thus assumed to be missing at random. Multiple imputations were conducted for the missing values based on the Markov Chain Monte Carlo method. The final results from the imputed data were based on 10 imputed data sets. Missing Fitbit step data and corresponding active minutes data were not in a monotonic pattern and thus assumed to be missing at random. Multiple imputations were conducted for the missing values based on the Markov Chain Monte Carlo method. The final results from the imputed data were based on 10 imputed data sets.
